# GPR125 positively regulates osteoclastogenesis potentially through AKT-NF-κB and MAPK signaling pathways

**DOI:** 10.7150/ijbs.70620

**Published:** 2022-03-06

**Authors:** Chen-Yi Tang, He Wang, Yan Zhang, Zhongliang Wang, Guochun Zhu, Abigail McVicar, Yi-Ping Li, Wei Chen

**Affiliations:** 1 Division in Cellular and Molecular Medicine, Department of Pathology and Laboratory Medicine, Tulane University School of Medicine, Tulane University, New Orleans, Louisiana, USA.; 2 Department of Pathology, School of Medicine, University of Alabama at Birmingham, Birmingham, Alabama, USA.; 3 Engineering Research Center of Molecular & Neuroimaging, Ministry of Education, School of Life Science and Technology, Xidian University, Xi'an, Shaanxi 710126, China.

**Keywords:** GPCR 125, Gpr125, osteoclasts, bone resorption, Akt signaling pathway, NFκB signaling pathway, MAPK signaling pathway

## Abstract

G-protein-coupled receptors (GPCRs) signaling is critical to cell differentiation and activation. However, the function of GPCRs in osteoclast differentiation and activation remains unclear. We found that the G-protein coupled receptor 125 (GPCR 125) gene (Gpr125) gene was highly expressed in osteoclasts through RNA-sequencing technology, qRT-PCR, and Western blot analysis. We characterized the role of GPCR125 in osteoclast differentiation and activation by loss-of-function and gain-of-function methods in osteoclasts. Osteoclasts with lentivirus-mediated GPR125 silencing demonstrated a dramatic reduction in differentiation and impaired bone resorption function. In contrast, overexpression of Gpr125 in osteoclasts increased NFATC1 expression and enhanced osteoclast differentiation and enhanced osteoclast-mediated bone resorption. These results indicated that GPCR125 positively regulates osteoclast formation and function. Following receptor activator of nuclear factor kappa-Β ligand (RANKL) stimulation, the expression levels of MAPK signaling pathway proteins phosphorylated-ERK (p-ERK) and phosphorylated-p38 (p-p38) were significantly decreased in the Gpr125 knockdown (sh-GPR125) group compared to its control group. We also found that phosphorylated AKT (p-AKT) expression was downregulated, as well as nuclear factor kappa-B (NF-κB) signaling pathway protein phosphorylated-IKB alpha (p-IKBα). Our results demonstrated that GPCR125 positively regulates osteoclasts via RANKL-stimulated MAPK and AKT-NF-κB signaling pathways, and GPCR125 could potentially be utilized as a novel therapeutic target in bone related diseases including osteoporosis.

## Introduction

Osteoclasts are unique multinucleated cells with the ability to resorb bone, and play an important role in bone remodeling 1. When osteoclast-mediated bone resorption is greater or lesser than osteoblast-mediated bone formation, the imbalance of bone growth and regulation can lead to metabolic bone diseases. Among the various types of metabolic bone diseases in adults, osteoporosis and osteopetrosis are the most common 2-4. For example, in the United States alone, 28 million adults are affected by osteoporosis and suffer from reduced bone mass and increased bone fractures 3. Therefore, the study of the developmental mechanism of osteoclasts is evidently important for the treatment of such prevalent diseases.

Osteoclasts are multinucleated giant cells localized in bone and are generated from hematopoietic stem cell lines derived from the monocyte-macrophage lineage 5. When induced by macrophage colony-stimulating factor (M-CSF, also called CSF-1) and receptor activator of nuclear factor kappa-B (RANK) ligand (RANKL), osteoclast progenitors proliferate and differentiate into mononuclear pre-osteoclasts. Then, after fusing with one another, pre-osteoclasts become multinucleated osteoclasts 5-7. The distinguished characteristics of multinucleated osteoclasts are its polarization (asymmetrical expression of proteins which allows attachment to and resorption of bone) and its secretion of specific protein markers such as Tartrate - resistant acid phosphatase 5 (TRAP) and Cathepsin K (CTSK) 8. In addition, osteoclasts organize its cytoskeleton to form an actin ring, which serves as a sealing zone to bone and a critical component of bone resorption. 9. Then, osteoclasts secrete protons into the resorption area to decrease the extracellular pH and degrade bone matrices 10. The above activities of osteoclasts can be used to characterize osteoclasts during osteoclastogenesis.

There are a plethora of G-protein coupled receptors (GPCRs) within the cell membrane of osteoclasts. GPCRs, the category of most intensively studied drug targets, make up nearly 34% of all drugs approved by the FDA 11, 12. To be a GPCR, two main requirements are needed. One is that 25 to 35 consecutive residues for the seven sequence stretches have a relatively high degree of calculated hydrophobicity, and the other is that the ability of the receptor to interact with a G-protein 13. The most frequently used classification system for GPCRs is to use clans A, B, C, D, E, and F, which cover all GPCRs, in both vertebrates and invertebrates, and subclans are divided by roman number nomenclature 14. GPCRs are seen as major therapeutic targets in various diseases: diabetes, obesity, respiratory and cardiovascular diseases, Alzheimer's disease, and CNS disorders 11, 15. For bone metabolism, GPR137b 16, GPR120 17 and OGR1 18, have been reported to regulate osteoclast differentiation and function. It also has been reported that GPCRs including Rs1^19^ and GRK [20]regulate osteoblast differentiation and bone formation. GPR125 is an orphan receptor, as well as Adhesion-G-Protein-Coupled Receptor A3 (ADGRA3) belonging to the Adhesion GPCRs (aGPCRs) family. This receptor plays an important role in tumor angiogenesis 21, 22 and was originally described as a spermatogonial stem cell marker 23. Moreover, GPR125 can modulate planar cell polarity signaling 24. In our previous research, we found that Gα13, which belongs to the G12 subfamily of the G-protein superfamily, negatively regulates osteoclast differentiation and activity through inhibition of the AKT-Gsk3β-NFATc1 signaling pathways 25. Therefore, GPCRs in osteoclasts can be potential targets in the treatment of osteoclast-related bone diseases 26.

In an RNA-seq analysis, we found that GPR125 is highly expressed in osteoclasts. Although GPR125 is highly expressed in bone, the effects of GPR125 on osteoclasts have not been studied. In this research, we examined the functions of GPR125 in osteoclast formation, differentiation, activation, survival, and acidification by lentivirus-mediated silencing and overexpression of *Gpr125* in osteoclast precursors. We also explored the mechanism underlying the role of GPR125 in osteoclasts. The activation of different downstream signaling pathways, such as NF-κB, AKT, ERK1/2, and p38 MAPK signaling pathways, by RANKL stimulation results in the increased expression of genes essential to osteoclast differentiation and in the expression of transcription factors for osteoclastogenesis 25. We detected RANKL-induced signaling pathway related protein changes in *Gpr125* knockdown cells and found significantly decreased levels of p-AKT in sh-GPR125 infected cells, as well as significantly decreased p-p65 and p-IKBα expressions in early stages of RANKL stimulation. Collectively, these data illustrated that GPR125 responds to RANKL stimulation to promote osteoclastogenesis through the upregulation of AKT and NF-κB signaling pathways to increase the expression of osteoclast genes. Our study suggests that targeting GPR125 may be a novel therapeutic approach for osteolytic diseases.

## Materials and Methods

### Ethics

The wild type (C57BL/6) mice used in the experiments were purchased from The Jackson Laboratory. All animal experiment was conducted according to the guidelines of the University of Alabama at Birmingham Institutional Animal Care and Use Committee (IACUC) and the Tulane University IACUC.

### Animal Experimentation

All animal experiments were performed according to the requirements and regulations of the Institutional Animal Care and Use Committee (IACUC) at the University of Alabama at Birmingham (UAB). In our study, all experiments used wild type (WT, C57Bl/6) mice that were purchased from The Jackson Laboratory.

### Reagents

All chemicals used during experimental procedures can be obtained from Sigma-Aldrich. Recombinant mouse M-CSF and RANKL were purchased from R&D Systems. Fetal Bovine Serum (FBS) was obtained from Hyclone Laboratories. α-Minimal essential medium (α-MEM) and Penicillin Streptomycin were from Gibco. TRIzol reagent was from Invitrogen. RevertAid RT Kit (K1691) and Fast SYBR Green Master Mix (4309155) were purchased from Thermo Scientific. Lentivirus titer test Kit (LV900) was obtained from ABM. Polybrene Infection / Transfection Reagent (TR-1003) was from Sigma-Aldrich. These antibodies (Mouse-anti-Ctsk, sc-48353; Rabbit-anti-Nfatc1, sc-13033; Goat-anti-ATP6i, sc-162300; Mouse-anti-PU.1, sc-390405; Mouse-anti-GAPDH, sc-47724;) were obtained from Santa Cruz Inc. These antibodies (Rabbit-anti-c-Fos, 2250; Rabbit-anti-IκBα, 4812; Rabbit-anti-phospho- IκBα(Ser32), 2859; Rabbit-anti-NF-κB p65(D14E12), 8242; Rabbit-anti-phospho- NF-κB p65(Ser536), 3033; Rabbit-anti-Akt, 4685; Rabbit-anti-phospho-Akt(Thr308), 2965; Rabbit-anti-GSK-3β, 12456; Rabbit-anti-phospho-GSK-3β(Ser9), 5558; Rabbit-anti-p44/42 MAPK(Erk1/2), 4695; Rabbit-anti-phospho-p44/42 MAPK(Erk1/2)(Thr202/Tyr204), 4370; Rabbit-anti-p38 MAPK, 9212; Rabbit-anti-phospho-p38 MAPK(Thr180/Tyr182), 4631; Rabbit-anti-JNK, 9252; Rabbit-phospho-SAPK/JNK(Thr183/Tyr185), 9251) were obtained from Cell Signaling Technology. The GPR125 polyclonal antibody (PA5-99891) was from Invitrogen. Horseradish peroxidase (HRP)-goat-anti-rabbit (7074) was from Cell Signaling Technology, HRP-rabbit-anti-mouse (A9044) was from Sigma, and HRP-donkey anti-goat (705-035-147) was from Jackson ImmunoResearch. FITC donkey-anti-mouse antibody and TRITC donkey anti-Goat antibody were from Thermo Scientific, and DAPI (4',6-diamidino-2-phenylindole) was from Sigma. The primer information is show in Table S1.

### Mouse bone marrows (MBMs) extraction and monocyte or osteoclast cell culture

Recombinant mouse macrophage-colony stimulating factor (M-CSF) and RANKL were purchased from R&D Systems, USA. Fetal Bovine Serum (FBS) was obtained from Hyclone Laboratories. Alpha-minimal essential medium (α-MEM) and Penicillin Streptomycin were acquired from Gibco. MBMs were isolated from the femurs of 8-week-old C57BL/6 mice and cultured as previously described 27-29. Firstly, collected MBM was cultured in α-MEM with 10% FBS containing 20 ng/ml M-CSF overnight. Adherent cell was primary monocytes. Non-adherent MBM was collected and seeded into 12-well plates (1×10^5^cell/well) or 24-well plates (5×10^4^cell/well), in wells or in bovine cortical bone slices, according to the experiment requirements. Then cells were cultured in α-MEM with 10% FBS containing M-CSF (10ng/mL) and RANKL (10ng/mL) for 5 days or 7 days continuously to induce osteoclasts.

### RNA-seq analysis

The total RNA was extracted from primary monocytes or osteoclasts induced for 3 days or 5 days with TRIzol reagent (Invitrogen Corp., Carlsbad, CA) reagent according to the manufacturer's protocol to perform RNA-sequence analysis. These RNA samples were sequenced on an Illumina HiSeq 2000 system (Illumina, San Diego, CA) 30 to obtain 100 base-pair single-end reads per sample 31. The modified results can be imported to Heatmapper web server to visualize the Heat map 32.

### Quantitative Reverse Transcription Polymerase Chain Reaction (qRT-PCR) analysis

The RNA extraction procedure was the same operation used in RNA-seq analysis. The total RNA was reverse transcribed into cDNA using RevertAid RT Kit (K1691, Thermo Scientific) according to the manufacturer's instructions. We carried out qPCRs by using SYBR Green Reagents (4309155, Thermo Scientific) and Step-One Real-Time PCR System (Life Technologies, Applied Biosystems) 30, 33. The expression of glyceraldehyde-3-phosphate dehydrogenase (*Gapdh*) was used as an endogenous control for normalization. The primer information is shown in **Table S1**.

### Immunofluorescence (IF) stain assay

The prepared femur sections of newborn C57BL/6 mice were subjected to antigen retrieval with heat-induced antigen retrieval solution (ab973, abcam). The specific IF stain procedure was performed as outlined previously 33. Primary antibodies, goat anti-GPR125 (1:200) (Santa Cruz) and mouse-anti-Ctsk (Santa Cruz, sc-48353) were applied to one section. After overnight incubation, secondary antibodies, TRITC donkey anti-Goat antibody (Thermo Scientific) and FITC donkey-anti-mouse antibody (Thermo Scientific) were used to show the specific expression location. Nuclei were visualized with DAPI (1ug/mL).

### Gpr125 gene knockdown or overexpression by lentivirus transduction

Five pLKO.1 vectors encoding short hairpin RNAs (shRNAs) targeting the mRNA of mouse *Gpr125* were purchased from Sigma. The detailed information was as follows: sh-Gpr125#1 (TRCN0000238969, Clone ID: XM_132089.7-4475s21c1); sh-GPR125#2 (TRCN0000238970, Clone ID: XM_132089.7-3818s21c1); sh-GPR125#3 (TRCN0000238973, Clone ID: XM_132089.7-716s21c1); sh-GPR125#4 (TRCN0000238972, Clone ID: XM_132089.7-3214s21c1), sh-GPR125#5 (TRCN0000238971, Clone ID: XM_132089.7-4039s21c1). A pLKO.1 vector encoding scramble shRNA sequence was also purchased as negative control (referred as 'sh-Sc' in the paper). These lentiviral sh-GPR125 can be used to achieve the knockdown of *Gpr125* gene expression in osteoclasts. One pLX304-GPR125 (Clone ID: TOLH-1510144) was obtained from CCSB-Broad ORF Lentiviral Expression Library of TransOMIC Technologies Inc. to achieve the overexpression of *Gpr125* in osteoclasts. Lentivirus was produced by co-transfecting with targeted plasmid, p CMV-dR8.2, p CMV-VSV-G and pLB-GFP, together into 293T cell line as previously described 28, 29. Then the supernatants were harvested 56-62 hours after transfection. The titer of these lentivirus was tested by the qPCR Lentivirus Titration (Titer) Kit (ABM, LV900) according the manufacturer's instructions 34. MBMs were transduced with lentivirus supernatants according to the experiment design in the presence of 8μg/μL polybrene for 24 hours on Day 0 (the first day when MBMs were induced by M-CSF/RANKL). The cell density of MBMs in 12-well plate was 1×10^5^cellls/cm^2^. The final Multiplicity of Infections (MOI) was controlled to 5 according the previous experiment. After incubation with lentivirus and polybrene for 24 hours, cells were cultured in normal medium with M-CSF/RANKL. After 4-5 days from culture, infected cells can be used for stain assay or expression analysis.

### Western blot analysis

Cells cultured with medium containing both M-CSF and RANKL for 5 days were lysed with lysis buffer according to protocol described previously 35, 36. These proteins were loaded onto 8% SDS-polyacrylamide gels. Primary antibodies against GPR125, NFATc1, C-Fos, PU.1 and GAPDH were used. For the phosphorylation assay, monocytes or macrophages (infected by lentivirus - sh-GPR125#2, cultured with medium containing M-CSF/ RANKL for 3 days) were starved for 4 hours and stimulated by RANKL (20ng/mL) for different times (0, 5, 10, 20, 40, 60 minutes). These proteins were performed by 10% SDS-polyacrylamide gels. These antibodies (Mouse-anti-Ctsk, sc4-8353; Rabbit-anti-Nfatc1, sc-13033; Goat-anti-Atp6i, sc-162300; Mouse-anti-Pu.1, sc-390405; Mouse-anti-GAPDH, sc-47724;) were obtained from Santa Cruz Inc. These antibodies (Rabbit-anti-c-Fos, 2250; Rabbit-anti-IκBα, 4812; Rabbit-anti-phospho-IκBα (Ser32), 2859; Rabbit-anti-NF-κB p65 (D14E12), 8242; Rabbit-anti-phospho-NF-κB p65 (Ser536), 3033; Rabbit-anti-Akt, 4685; Rabbit-anti-phospho-Akt (Thr308), 2965; Rabbit-anti-GSK-3β, 12456; Rabbit-anti-phospho-GSK-3β (Ser9), 5558; Rabbit-anti-p44/42 MAPK (Erk1/2), 4695; Rabbit-anti-phospho-p44/42 MAPK(Erk1/2) (Thr202/Tyr204), 4370; Rabbit-anti-p38 MAPK, 9212; Rabbit-anti-phospho-p38 MAPK (Thr180/Tyr182), 4631; Rabbit-anti-JNK, 9252; Rabbit-phospho-SAPK/JNK(Thr183/Tyr185), 9251) were obtained from Cell Signaling Technology. Horseradish peroxidase (HRP)-goat-anti-rabbit (7074) was from Cell Signaling Technology, HRP-rabbit-anti-mouse (A9044) was from Sigma, and HRP-donkey anti-goat (705-035-147) was from Jackson ImmunoResearch. Member images were captured by Fluor-S-Multi-Imager, then results quantified by Image J software from the National Institutes of Health 37. Results were normalized by the housekeeping protein Tubulin or Gapdh.

### Trap stain assay

TRAP (Tartrate - resistant acid phosphatase 5) staining was performed by a leukocyte acid phosphatase kit (Sigma, 387-A) to examine osteoclasts size, formation and activity as described 25, 38.

### WGA stain assay

To detect the bone resorption function of osteoclast, MBMs (5×10^4^) were seeded on bovine cortical bone slides in 24-well plates and transduced with different viruses. After 7 days of culture, these slices were harvested to perform the wheat germ agglutinin (WGA) stain as previously described 39. The cells on slices were removed by sonication in phosphate buffer solution (PBS) then performed with 3% H_2_O_2_ for 10 minutes. Next, the slices were stained with Peroxidase-conjugated WGA-lectin Kit (Sigma-Aldrich, L-3892) according the manufacture's protocol. When proceeded with DAB Peroxidase (horseradish peroxidase [HRP]) Substrate Kit (Vector Laboratories, SK-4100), resorption pit will be stained brown 40.

### F-actin ring stain assay

To detect the polarization and resorptive activity, we performed the F-actin ring stain as previously described 27. MBMs were cultured directly on the 24-well plates or the bovine cortical bone slices in the 24-well plates. Then cells were cultured with medium containing M-CSF/RANKL for 5 days. Cells were fixed with 3.7% formaldehyde for 15 min, washed by cold PBS 3 times, and then permeabilized in 0.2% Triton X-100 for 10 min at room temperature. Next, cells were blocked with 1% goat serum and incubated with rhodamine phalloidin (Life Technologies, USA) and primary antibody-mouse-anti-Ctsk (Santa Cruz, sc-48353) overnight. The following day, cells were then rinsed with PBS and incubated with FITC anti-mouse IgG secondary antibody (Thermo Scientific) for 90 min. Lastly, the nuclei were stained with DAPI (1µg/mL, Sigma). The stained cells were visualized under a fluorescence microscope (Leica Texas Red filter) 30. For quantification of IF stain, we used NIH Image J to perform counts.

### Acridine orange stain assay

The acid secretion function of osteoclasts was determined by Acridine orange (AO) stain as previously described 41. Osteoclasts cultured for 5 days were stained in α-MEM containing 5 μg/mL of Acridine orange (Sigma) for 15 min at 37℃, washed with PBS, and observed under a fluorescence microscope (Leica Texas Red filter) with a 490 nm excitation filter and a 525 nm arrest filter 29.

### Statistical analysis

All experimental data are presented as mean ± SEM of triplicate independent samples. The significant differences between two groups were analyzed with Student's *t*-test with GraphPad Prism (GraphPad Software, Inc., USA) statistical program, version 8.1, considering *P* value as follows: * *p* < 0.05, ** *p* < 0.01, *** *p* < 0.001 **** *p* < 0.0001. ns, no significant difference. Error bars depict SEM. Data represented in figures.

## Results

### *Gpr125* is highly expressed in osteoclasts *in vitro* and *in vivo*

**Figure 1:**
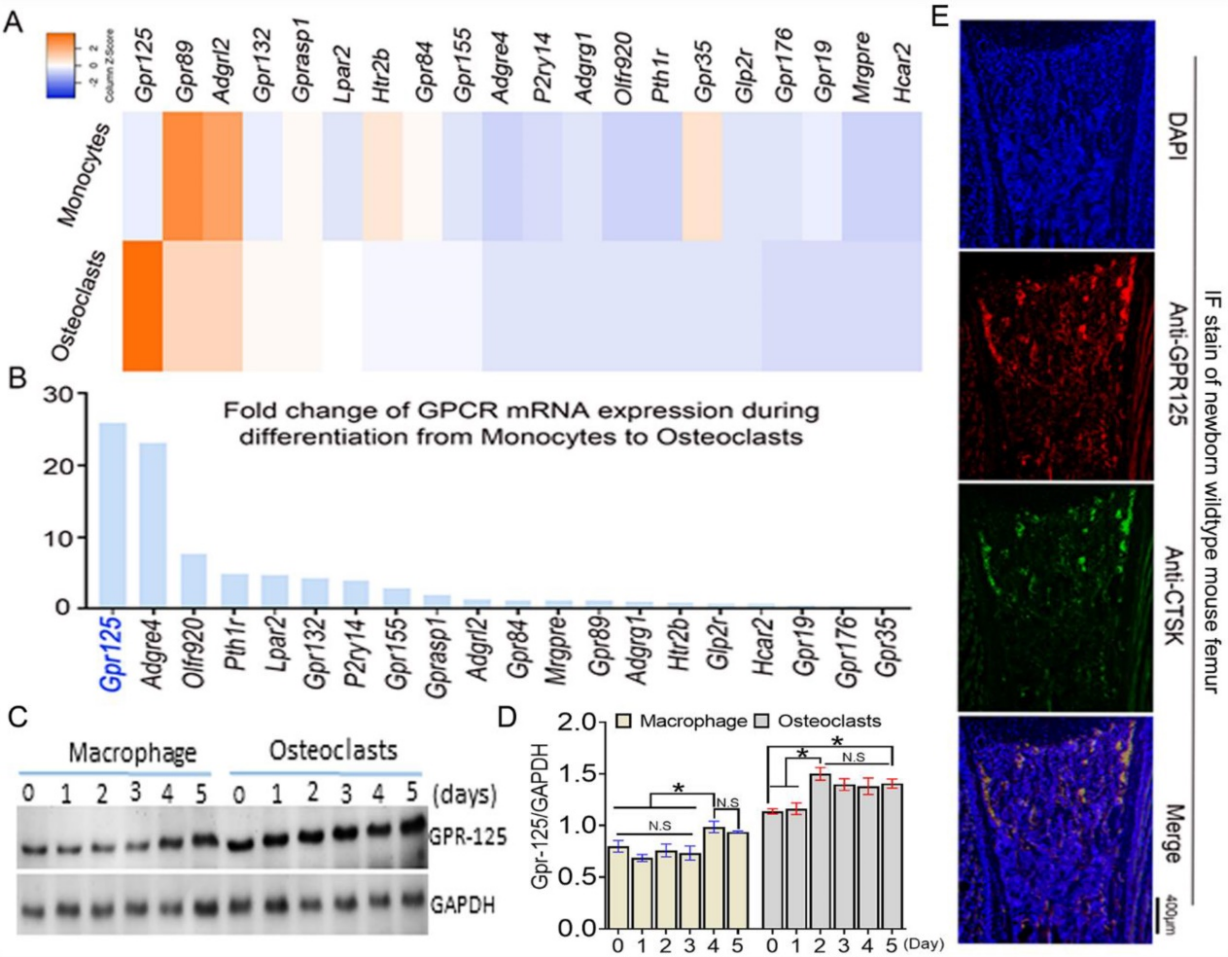
***Gpr125* is expressed in mice osteoclasts *in vitro* and *in vivo* and increases during the differentiation from monocytes to osteoclasts. (A)** Heatmap analysis of 20 GPCR mRNA expression levels in monocytes and osteoclasts by RNA-seq analysis. Orange denotes high expression; blue denotes low expression. **(B)** Column chart analysis of the fold change of 20 GPCR mRNA expression levels when cells differentiated from monocytes to osteoclasts from RNA-seq analysis. **(C)** Protein level of GPR125 in macrophages and osteoclasts induced for 5 days. **(D)** Quantification of C. **(E)** *Gpr125* expression in osteoclast of the femur sample of newborn mouse *in vivo* showed by IF stain with anti-Gpr125 (red), anti-Ctsk (green) and DAPI (blue). Scale bar, 400μm. Bars on graphs represent mean ± SEM, * *p* < 0.05. ns, not significant.

To explore the function of GPCRs in osteoclastogenesis, we performed genome-wide expression analysis using WT murine bone marrow monocytes (MBMs) and osteoclasts induced by M-CSF and RANKL. In the RNA-seq analysis of monocytes (with M-CSF stimulation) and osteoclasts (with M-CSF and RANKL double stimulation) derived from the same source wide-type mice, we analyzed 20 GPCR-related gene expression at mRNA levels. The results of genome-wide expression analysis showed several GPCR-related genes highly expressed in osteoclasts compared with others (**Fig.1A**). We compared the expression level fold change of 20 GPCR-related genes in monocytes and osteoclasts (**Fig. 1B**). GPR125 showed the greatest increase during osteoclast differentiation induced by RANKL, with a fold change increase of more than 25 following differentiation from monocytes to osteoclasts. This result indicates that GPR125 may have a role in osteoclastogenesis*.* Furthermore, we carried out western blot to confirm the protein levels of *Gpr125* in monocytes and osteoclasts (**Fig. 1C, D**). Notably, in protein levels, the GPR125 expression was obviously increased in both macrophages and osteoclasts as the induction days increased (**Fig. 1C, D**). Furthermore, GPR125 expression in osteoclasts was higher than in macrophages as the induction days increased (**Fig. 1C, D**). At day 0, the protein levels of GPR-125 were 1.7-fold higher in osteoclasts compared to macrophages, and by day 2 the protein levels of GPR-125 were 2-fold higher in osteoclasts compared to macrophages (**Fig. 1C, D).** In addition, we also explored the expression level of *Gpr125* in osteoclasts* in vivo* by immunofluorescence (IF) stain of femur tissue of newborn wild-type mice. GPR125 (red) partially merged with Cstk (green), a marker of mature osteoclasts, further indicating that *Gpr125* colocalizes with Ctsk and is expressed in osteoclasts (**Fig. 1E**). These results demonstrated that GPR125 was highly expressed *in vitro* culture of osteoclasts compared to macrophages, and *in vivo* by IF stain, suggesting a role of GPR125 in osteoclasts.

### The knockdown of *Gpr125* inhibits osteoclast differentiation and function

**Figure 2:**
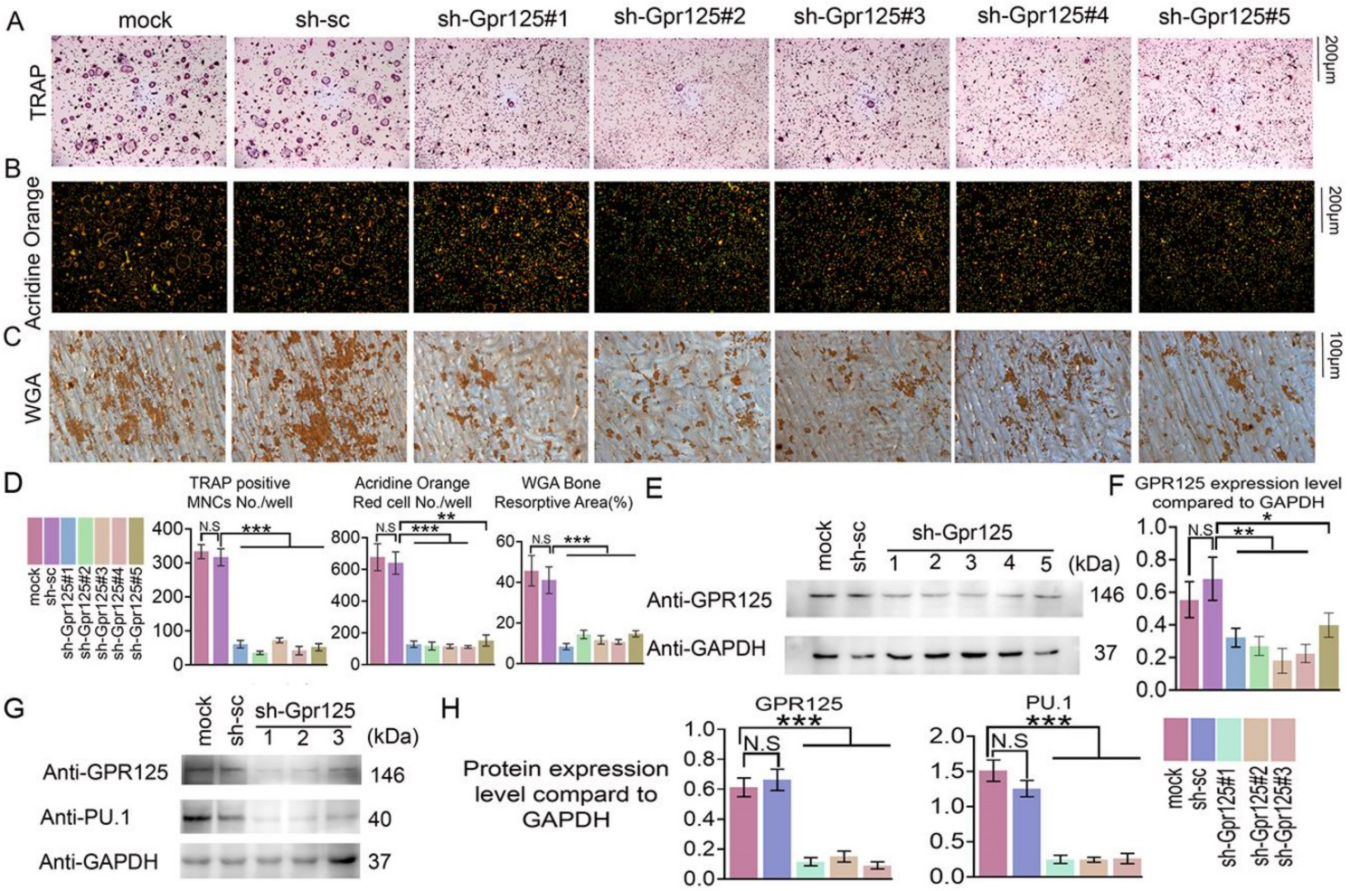
** Knockdown of *Gpr125* impairs osteoclast differentiation and function. (A-G)** MBM was transduced by sh-SC, sh-GPR125 lentivirus, or mock control and stimulated by M-CSF (10ng/mL) and RANKL (10ng/mL). **(A)** When cells were induced by M-CSF/RANKL for 5 days, cells were stained for TRAP activity at a 12-well-plate. Scale bar = 200μm. **(B)** To detect the acid secretion function, cells were performed with Acridine Orange (AO) stain in a 24-well-plate when induced by M-CSF/ RANKL for 5 days. Scale bar = 200μm.** (C)** To determine the bone resorption activity of mature osteoclasts, cells of three groups were stained with WGA reagent. Scale bar = 100μm. **(D)** Quantification of **A-C**. **(E)** Western blot analysis of osteoclasts from three groups on Day 5 to confirm the knockdown of *Gpr125*. **(F)** Quantification of protein levels of Gpr125 in E normalized to Gapdh. **(G)** Western blot analysis of osteoclasts from three groups on Day 5 to explore the influence of the knockdown of Gpr125 on the expression of osteoclast marker protein Pu.1. **(H)** Quantification of protein levels of Gpr125 and Pu.1 in G normalized to Gapdh. Data is expressed as mean ± SEM, one dot represents one sample (n = 3). * *p* < 0.05, ** *p* < 0.01, *** *p* < 0.001.

To determine the functions of GPR125 during osteoclastogenesis, we silenced *Gpr125* expression by applying sh-GPR125 lentivirus. Short hairpin RNA (shRNA) can silence gene expression mediated by the lentivirus system. Five lentiviruses encoding shRNA targeting *Gpr125* mRNA (using pLKO.1-shRNA-Gpr125) were produced by 293T package system. Packaged lentivirus was transduced into MBM on Day 0. Then, these cells were cultured in M-CSF/ RANKL for 5 days. After lentivirus preparation, we added pLB-GFP plasmid DNA to perform the co-transfection. The *Gpr125* expression of sh-GPR125 group decreased compared to sh-SC group or mock group (sh-GPR125 group: MBM transduced with sh-GPR125 lentivirus; sh-SC group: MBM transduced with sh-scramble lentivirus; mock group: MBM without lentivirus). Western blot was used to confirm successful silencing of GPR125 **(Fig 2E, F).** The TRAP stain results showed decreased osteoclasts number and size following *Gpr125* silencing (**Fig. 2A, D**), as well as a 7-fold decrease in TRAP+ osteoclasts/well in sh-Gpr125 treated cells **(Fig 2D)**, illustrating a potential role of *Gpr125* to positively regulate osteoclast formation. To determine the influence of *Gpr125* on the acid secretion function of osteoclasts, Acridine Orange (AO) stain was carried out to experimental and control groups. The mature osteoclasts in sh-GPR125 group showed less acidification levels than the mock control group, with 13-fold fewer AO red cells/well (**Fig. 2B, D**). We also explored the bone resorption pits by WGA stain. Compared to the control group, the resorption pit was decreased by 2.7-fold in sh-GPR125 group osteoclasts (**Fig. 2C, D**). The above data showed that GPR125 can positively affect both the formation of osteoclasts and their function. Moreover, we further detected the osteoclastogenesis marker expression of Pu.1 to confirm the effect of *Gpr125* knockdown at the protein level in osteoclasts. The level of Pu.1 decreased in sh-GPR125#1 group by 6-fold compared to the mock control, while the level of Gpr125 was downregulated by 6-fold in sh-GPR125#1 group compared to mock control (**Fig. 2G, H**). These data indicated that *Gpr125* deficiency inhibits osteoclast formation and function, and decreases the protein levels of key osteoclast genes *in vivo*.

### The knockdown of *Gpr125* inhibits F-actin ring formation and osteoclastogenesis-related gene expression at the mRNA level

**Figure 3:**
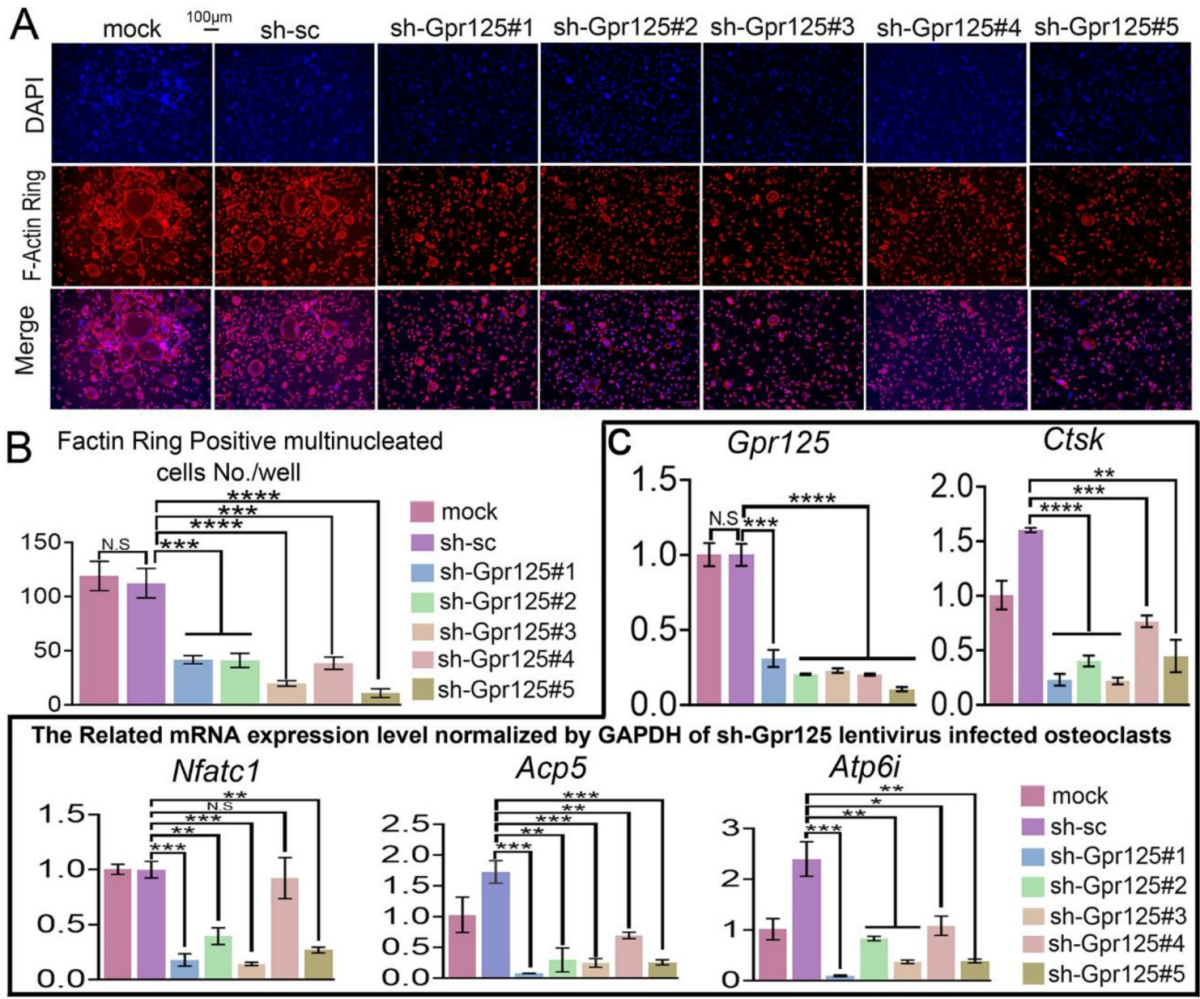
** Knockdown of *Gpr125* inhibits F-Actin ring formation and decreases osteoclastogenesis-related genes expressions. (A-C)** MBMs infected by different lentivirus respectively and induced by M-CSF (10 ng/mL) and RANKL (10 ng/mL). **(A)** To detect the influence of *Gpr125* knockdown on F-Actin ring formation, cells cultured for 5 days were conducted with F-Actin ring stain. DAPI (blue), F-Actin (red), merge of F-Actin and DAPI (purple). Scale bar = 100μm. **(B)** Quantification of F-actin ring positive cells in A. **(C)** qRT-PCR of the sh-GPR125 treated osteoclasts to detect mRNA expression levels of *Gpr125*, *Ctsk*, *Nfatc1*, *Acp5*, and *Atp6i* compared with *GAPDH*. One dot represents one sample (n = 3). * *p* < 0.05, ** *p* < 0.01, *** *p* < 0.001, **** *p* < 0.0001. ns, no significance.

As an important structure for osteoclast maturation, F-Actin rings can be detected by F-Actin ring stain in mature osteoclasts 42. To explore how GPR125 regulates F-Actin ring formation, we carried out F-Actin ring stain to cells transfected with lentiviral sh-GPR125. When cells were cultured in wells, there were less multinucleated osteoclasts with a complete F-actin ring in the sh-GPR125 group compared to its control group. The morphological characteristic indicates that GPR125 promotes F-Actin ring formation. We also seeded cells in bone slides and operated the same cell culture. Co-dyeing of F-Actin ring and anti-Ctsk were performed after cells had been cultured for 5 days. In sh-GPR125 group, mature osteoclasts F-Actin ring formation was decreased, and *Ctsk* expression level was downregulated (**Fig. 3A, B**). These results demonstrated that GPR125 positively regulates F-Actin ring formation and *Ctsk* expression in osteoclasts *in vitro* and *in vivo*. To further investigate the effects of loss-of-function of GPR125 on osteoclastogenesis-related genes, MBMs were transfected with lentiviral sh-GPR125 and sh-SC, respectively. After the cells were cultured for 5 days, we collected RNA and carried out qRT-PCR experiments. The results showed that GPR125 was downregulated in sh-GPR125 group more than 70% compared to its control group (**Fig. 3C**). Then we detected the osteoclastogenesis-related genes (*Ctsk*, *Nfatc1*, *Acp5*, *Atp6i*) expressions at the mRNA level in sh-GPR125 group. Compared to sh-SC control, the expression levels of Ctsk, Nfatc1, Acp5, and Atp6i were decreased by 4-fold, 5-fold, 4-fold, and 2-fold, respectively in sh-GPR125 group **(Fig. 3C)**. These results showed significantly decreased osteoclast gene expressions with the deficiency of *Gpr125*.

### The overexpression of *Gpr125* increases osteoclastogenesis-related gene expressions in mRNA level

**Figure 4:**
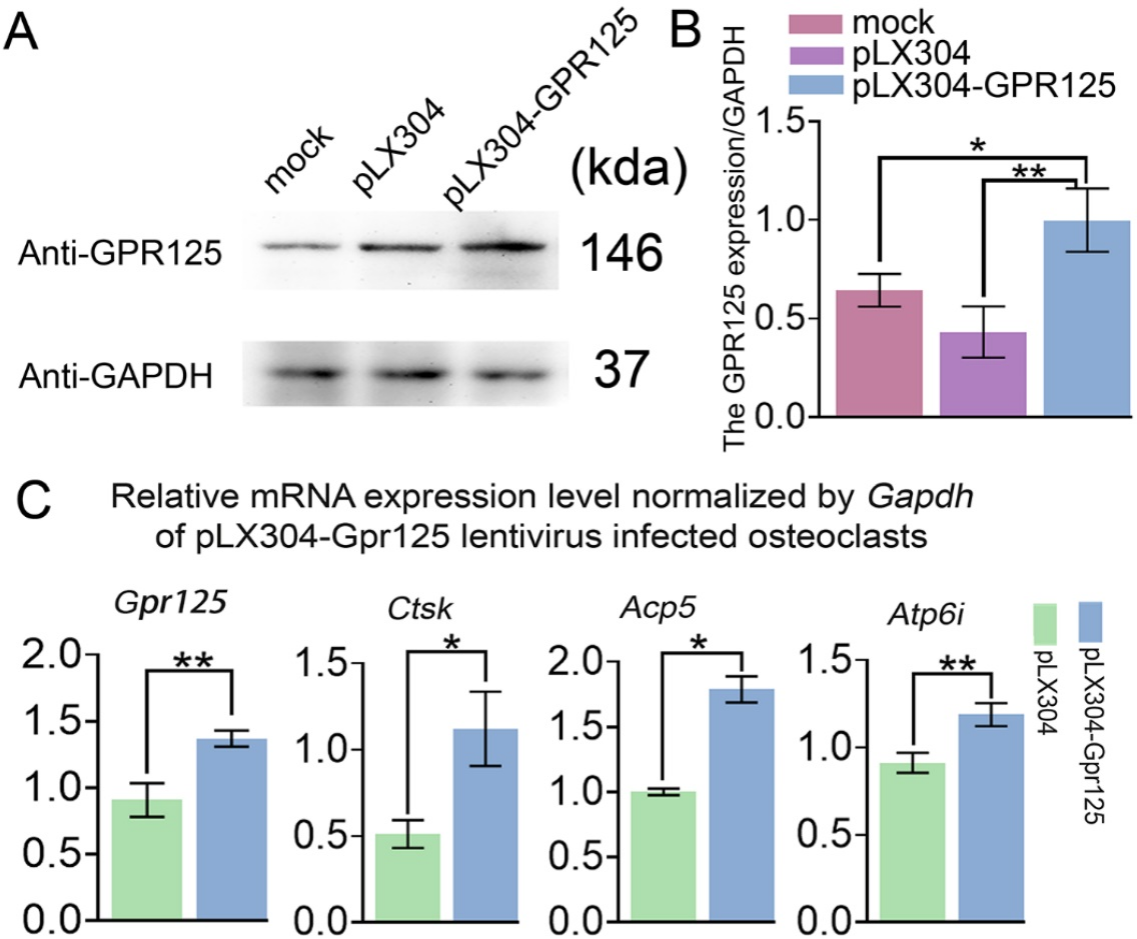
** Overexpression of *Gpr125* increases osteoclastogenesis related gene expression at the mRNA level. (A)** Western blot analysis confirming the overexpression of *Gpr125* after cells were transfected with pLX304-GPR125. **(B)** The quantification of data of (A). **(C)** qRT-PCR results of the pLX304-GPR125 infected osteoclasts. When cells were cultured for 5 days, qRT-PCR was carried out to measure the mRNA expression levels of *Gpr125*, *Ctsk*, *Acp5*, and *Atp6i* normalized by *GAPDH*. * *p* < 0.05, ** *p* < 0.01.

To examine the gain-of-function effects of GPR125 in osteoclastogenesis, MBMs were transfected with pLX304-GPR125 lentivirus to achieve the overexpression of *Gpr125* in osteoclasts. Western blot analysis was used to confirm *Gpr125* overexpression, and results showed increased GPR125 protein expression in the pLX304-GPR125 group compared to both pLX304 and mock groups (**Fig. 4A**). After quantification of Western blot analysis, we discovered significantly increased GPR125 expression normalized to GAPDH in the pLX304-GPR125 group compared to others (**Fig. 4B**). Specifically, osteoclasts with *Gpr125* overexpression had a 2-fold increase in GPR125 expression compared to the pLX304 group (**Fig. 4B**). Thus, *Gpr125* was successfully overexpressed in pLX304-GPR125 lentivirus infected osteoclasts. Then we performed qRT-PCR to examine the changes in mRNA expression levels of *Gpr125*, as well as osteoclast-related genes *Ctsk*, *Acp5*, and *Atp6i*. Results showed a 1.5-fold increase in *Gpr125* and significantly increased osteoclast-related gene expressions in the pLX304-GPR125 group compared to the pLX304 group (**Fig. 4C**). Specifically, *Ctsk* exhibited a 2.2-fold increase, *Acp5* showed a 1.7-fold increase, and *Atp6i* had a 1.3-fold increase in mRNA expression in pLX304-GPR125 lentivirus infected osteoclasts compared to pLX304 lentivirus infected osteoclasts (**Fig. 4C**). Therefore, *Gpr125* overexpression significantly increased osteoclast-related genes mRNA expressions, providing evidence that GPR125 may positively regulate osteoclastogenesis.

### The overexpression of *Gpr125* promotes osteoclast differentiation and function

**Figure 5:**
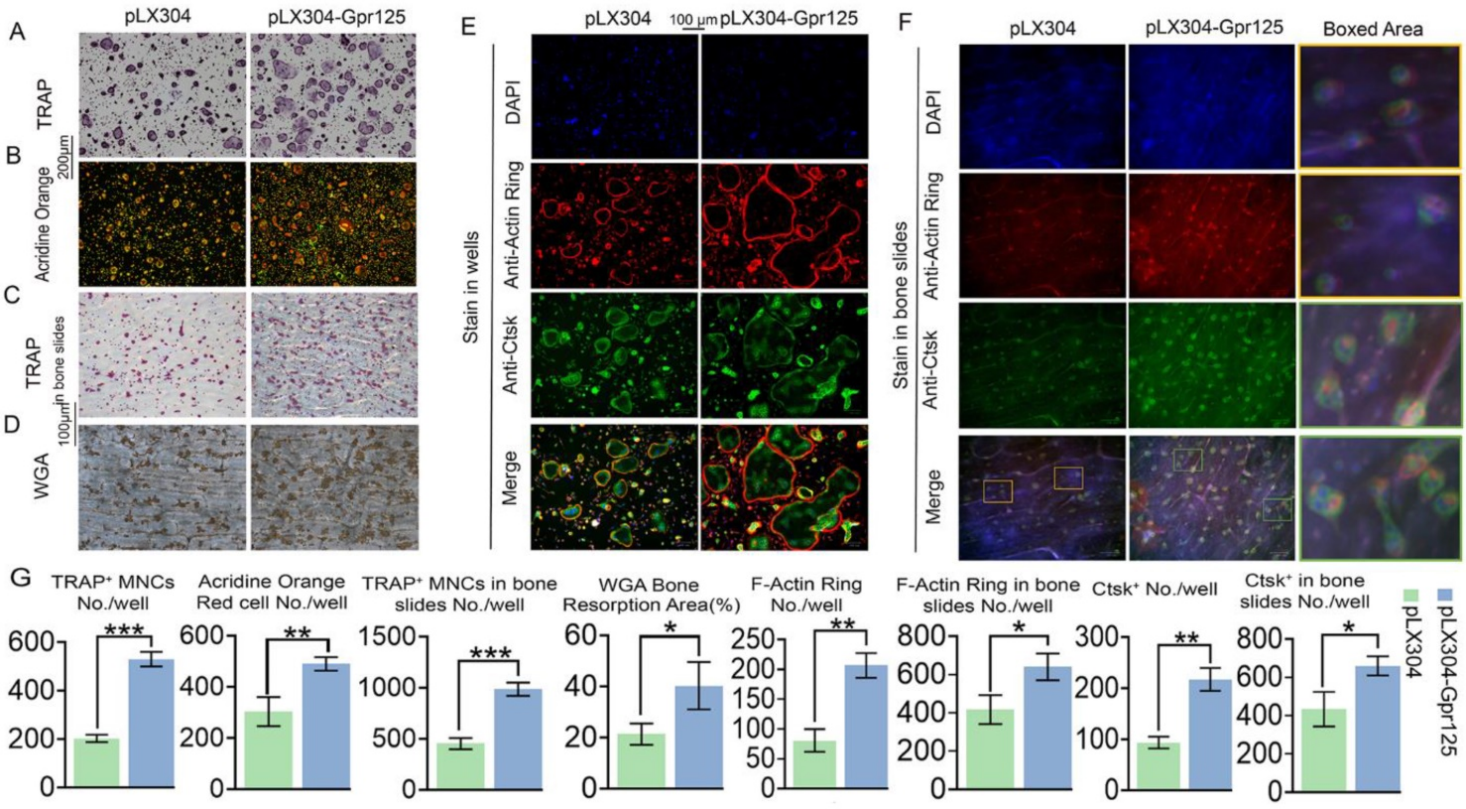
** Overexpression of *Gpr125* promotes osteoclast differentiation and function. (A-D)** Overexpression of *Gpr125* increases osteoclast differentiation. **(A-G)** MBMs were transduced by pLX304 or pLX304-GPR125 lentiviruses, and cells were stimulated by M-CSF (10 ng/mL) and RANKL (10 ng/mL). **(A)** When cells were cultured for 5 days, TRAP stain was performed to show TRAP activity. Scale bar = 200 μm. **(B)** After inducing cells by both M-CSF and RANKL for 5 days, AO stain was conducted to detect their acid secretion performance. Scale bar = 200 μm.** (C)** Cells seeded in bone slides were cultured for 5 days and underwent TRAP stain to show their TRAP activity when grown physiologically. Scale bar = 100 μm. **(D)** WGA stain was conducted to determine bone resorption activity when cells were cultured in bone slides for 7 days. Scale bar = 100 μm. **(E-F)** To detect the F-Actin ring formation and *Ctsk* expression levels at the same time, cells were seeded into wells **(E)** and bone slides **(F)** then cultured for 5 days. F-Actin stain and anti-Ctsk IF stain were performed simultaneously. DAPI (blue), F-Actin ring (red), anti-Ctsk (green), and merge region (orange). The frame region in merge picture of each group was shown in the boxed area. **(G)** Quantification of **(A-F)**. TRAP positive MNCs (Multinucleated cells) (≥ 3 nuclei). One dot represents one sample (n = 3). * *p* < 0.05, ** *p* < 0.01, *** *p* < 0.001.

To further examine the effects of GPR125 overexpression, we conducted various staining experiments. After performing TRAP stain, we discovered that GPR125 overexpression increased TRAP activity, with 2.5-fold increase of TRAP+ MNCs/well, as well as the size and quantity of osteoclasts (**Fig. 5A, G**). Moreover, AO stain was performed to detect the effect of GPR125 overexpression on osteoclast extracellular acidification. The staining results showed 1.6-fold more acid secretion in the pLX304-GPR125 group compared to the pLX304 group (**Fig. 5B, G**). Furthermore, we explored the physiologic TRAP activity by seeding cells on bovine cortical bone slides. In bone slides, there were 2-fold more osteoclasts in the pLX304-GPR125 group (MBM transfected with lentiviral pLX304-GPR125) than in the pLX304 group (MBM transfected with lentiviral pLX304), thus the polarization performance of osteoclasts increased in the pLX304-Gpr125 group (**Fig. 5C, G**). In addition, we performed WGA stain when cells were grown in bone slides for 7 days. The bone resorption ability in the pLX304-GPR125 group was greater than in the pLX304 group. The ratio of bone resorption area versus total bone slides was increased by more than 2-fold in the pLX304-GPR125 group (**Fig. 5D, G**). Also, when stained in wells, osteoclasts in the pLX304-GPR125 group exhibited thicker and larger F-actin rings than in the pLX304 group, with a 2.6-fold increase in F-actin rings/well (**Fig. 5E, G**). We also detected *Ctsk* expression by IF staining concurrently with F-Actin ring stain. *Ctsk* was mainly expressed in the cytoplasm and around the nucleus of osteoclasts. The *Gpr125* overexpression increased *Ctsk* (green) expression by more than 2.2-fold (**Fig. 5G, E**). Further, for physiologic F-Actin rings, osteoclasts were also grown on bone slides, and co-staining of F-Actin ring stain and anti-*Ctsk* IF stain were also carried out. There was 1.7-fold more F-Actin ring formation and 1.5-fold more *Ctsk* expression in the pLX304-GPR125 group (**Fig. 5F, G**). In the magnification image, we can examine how the F-Actin ring (red) and anti-Ctsk (green) stains in the pLX304-GPR125 were brighter when compared with the control group (**Fig. 5F**). This result also confirmed that GPR125 can affect bone resorption activity because it can affect two key factors of bone resorption: F-Actin ring formation and *Ctsk* expression. The above data showed that gain-of-function of *Gpr125* promotes the osteoclastogenesis in terms of osteoclast size, number, TRAP activity, acid secretion, F-Actin ring formation, *Ctsk* expression, and bone resorption, demonstrating an important role of Gpr125 in osteoclastogenesis.

### GPR125 positively regulates osteoclastogenesis via RANKL induced MAPK signaling pathways

**Figure 6:**
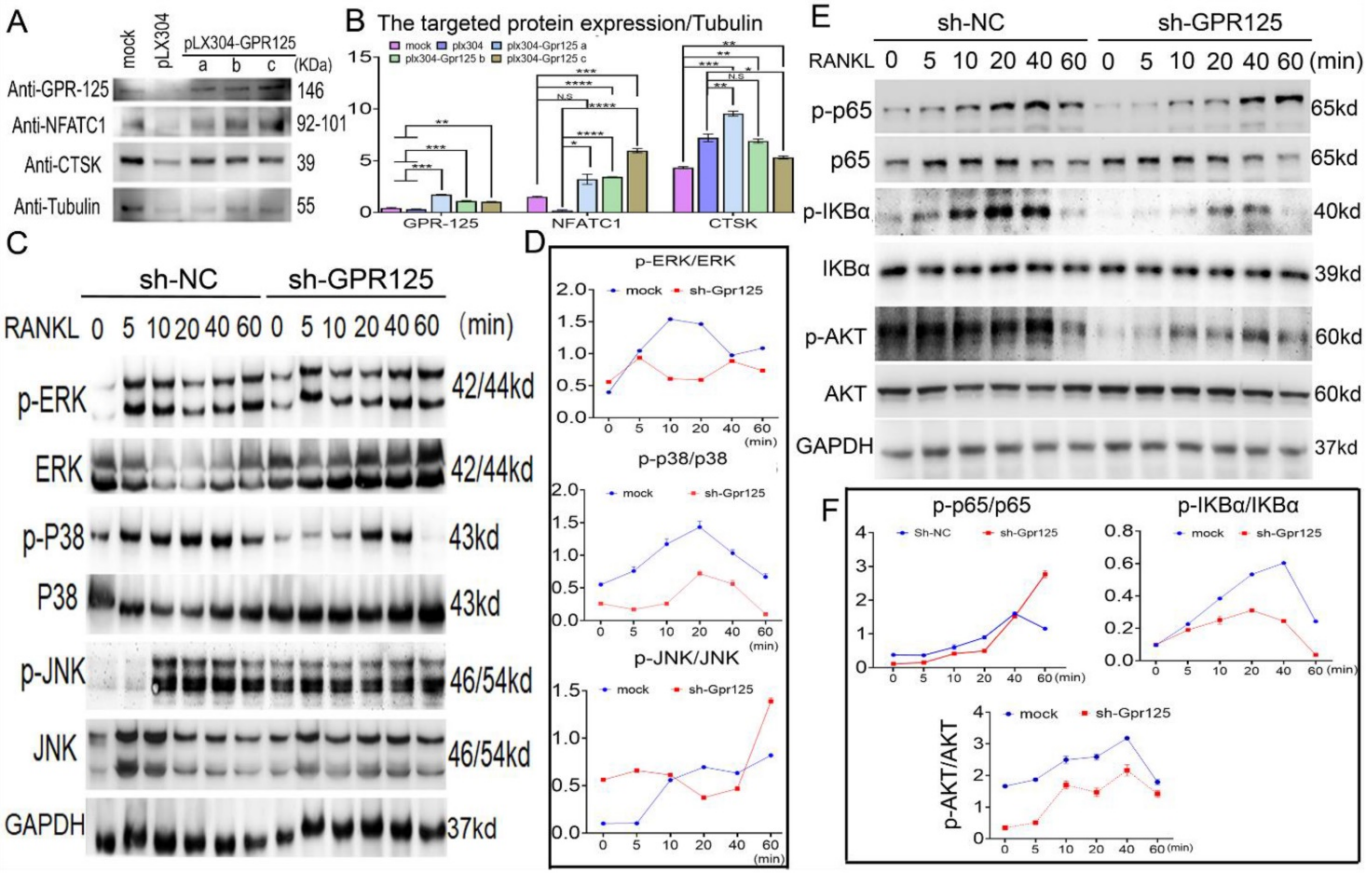
** Gpr125 promotes osteoclastogenesis via NF-κB and MAPK signaling pathways. (A)** MBMs infected by pLX304 or pLX304-GPR125 viruses, respectively, were continuously stimulated by M-CSF (10ng/mL)/RANKL (10ng/mL) for 5 days. Western blot analysis was used to confirm the overexpression efficiency of *Gpr125* and to detect the expression levels of NFATc1, CTSK, using Tubulin as control. **(B)** Quantification data of **(A)**. Protein expression levels were normalized by *GAPDH*. **(C)** Western blot analysis was used to explore the change of the phosphorylation of ERK, JNK, and p38 between sh-GPR125 and sh-NC groups, using *GAPDH* as the control. These cells were stimulated by RANKL (20ng/mL) when induced by M-CSF (10ng/mL) and RANKL (10ng/mL) for 3 days. **(D)** Quantification data of **(C)**. **(E)** Western blot analysis was used to explore the change of the phosphorylation of p65, IKBα, and AKT between sh-GPR125 and sh-NC groups. **(F)** Quantification data of **(E)**. The phosphorylation levels were shown by phosphor-protein level versus total protein level. One dot represents one sample (n = 3). * *p* < 0.05, ** *p* < 0.01, *** *p* < 0.001, **** *p* < 0.0001. ns, no significance.

To further investigate the mechanisms underlying the role of Gpr125 in osteoclasts, we examined the levels of osteoclast marker proteins NFATc1 and Ctsk. After confirming the overexpression of *Gpr125*, we quantified the expression levels compared to the mock group and found a 2-fold increase in the protein levels of both NFATc1 and Ctsk (**Fig. 6A, B**). To further investigate the mechanism by which GPR125 regulates osteoclastogenesis, we transfected osteoclasts with either sh-GPR125 or sh-SC (control group) and continuously induced cells by M-CSF/RANKL stimulation for 5 days. After starving cells and stimulating by RANKL, we harvested these proteins for Western blot analysis. Results showed that MAPK signaling pathway proteins p-p38 and p-ERK levels decreased during RANKL stimulation in sh-GPR125 group compared to its control group, but the p-JNK expression showed no significant difference (**Fig. 6C, D**). We also detected NF-κB signaling pathway proteins levels. We found that p-AKT, p-IKBα, and p-P65 expression levels were significantly decreased in the sh-GPR125 group (**Fig. 6E, F**). These data suggest a possible mechanism by which GPR125 promotes osteoclastogenesis by regulating AKT-NF-κB signaling pathways, as well as regulating MAPK signaling pathways.

## Discussion

**Figure 7:**
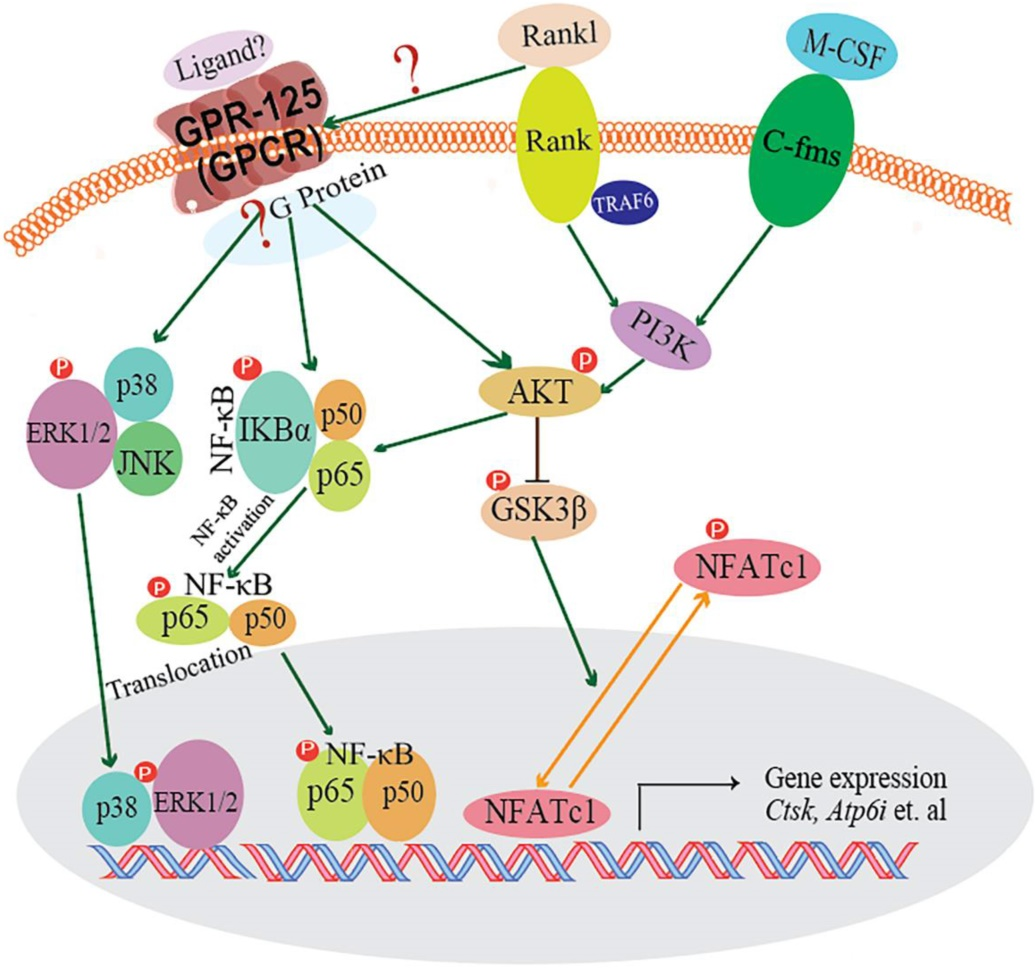
** The working model of how GPR125 positively regulates osteoclastogenesis.** GPR125 may positively regulate osteoclast differentiation through the RANKL-induced MAPK and AKT-NF-κB pathways to regulate the osteoclastogenesis-related transcriptional process and marker gene expression.

In our research, we demonstrated that GPR125 is highly expressed in osteoclasts. We explored the role of GPR125 by both loss-of-function and gain-of-function methods in osteoclasts. After transfection with sh-GPR125 lentivirus, mature osteoclasts demonstrated a dramatic reduction in differentiation and size, while osteoclast function was impaired. Corresponding to these phenotypes, overexpression of *Gpr125* in osteoclasts promoted osteoclast development and enhanced osteoclast-mediated bone resorption. These results indicated that GPR125 positively regulates osteoclast formation and function. In addition, we explored NF-κB signaling pathway proteins levels, and we found that p-AKT, p-IKBα, and p-P65 protein levels were significantly decreased after Gpr125 silencing. We further proposed that GPR125 increases osteoclast marker gene expression levels and promotes osteoclast formation by responding to RANKL stimulation to upregulate MAPK (MAPK-ERK and MAPK-p38) and AKT-NF-κB (p-IKBα and p-P65) signaling pathways (**Fig. 7**). Furthermore, we demonstrated that GPR125 is a positive regulator of osteoclastogenesis.

By RNA-seq analysis, we found that *Gpr125* is highly expressed in osteoclasts following the differentiation from macrophages to osteoclasts. We confirmed this finding by examination of the mRNA and protein levels in mature osteoclasts. GPR125 is a transmembrane signal transducer and belongs to the family of adhesion G-protein-coupled receptors 13. GPR125 has been postulated to play a crucial role in cell adhesion and signal transduction 43. Recent studies have demonstrated that Gpr125 inhibits the Wnt/β-Catenin signaling pathway 43. Interestingly, our study has highlighted that GPR125 may play an important role during osteoclastogenesis. The qRT-PCR results of *Gpr125* expression in monocytes and osteoclasts confirmed the RNA-seq analysis. Moreover, the anti-Gpr125 and anti-Ctsk co-IF stain in mice femur samples determined that GPR125 was expressed in osteoclasts *in vivo* (**Fig. 1**). To explore how GPR125 influences osteoclastogenesis, we used loss-of-function and gain-of-function approaches. We found that *Gpr125* knockdown not only inhibits osteoclast formation, cell size, and number but also decreases osteoclast function, such as acid secretion and bone resorption activity (**Fig. 2**). F-Actin ring, the indicator of correct osteoclast polarization and absorptive activity 27, 44, is an important cytoskeleton structure. Whether grown in a well plate or bovine cortical bone slices, *Gpr125* knockdown suppressed F-Actin ring formation (**Fig. 3**). In addition, the effect of *Gpr125* knockdown did not solely effect osteoclast morphology. The osteoclastogenesis-related genes decreased as *Gpr125* expression decreased at mRNA and protein levels (**Fig. 2, 3**). On the other hand, the overexpression of *Gpr125* showed the opposite result of *Gpr125* knockdown (**Fig. 4, 5**). Hence, our results reveal that GPR125 enhances osteoclasts differentiation and activity.

RANK-RANKL signaling pathway plays a pivotal role in osteoclastogenesis. When RANKL binds to RANK, TRAF6 is recruited, and downstream signaling pathways are activated to stimulate osteoclast differentiation and functions. The downstream signaling pathways are known as: NF-κB,AKT-Gsk3β,MAPKs (ERK, p38), etc. Previous research by our group and others has demonstrated that Akt induces osteoclast differentiation through inhibiting the GSK3β signaling cascade, which can regulate NFATc1 translocation from the cytoplasm into the nucleus 25, 45. GPR125 is one member of adhesion G protein-coupled receptors (aGPCRs), which consists of transmembrane-spanning regions merging with one or several functional domains with adhesion-like motifs in the N terminus. The N termini are variable in length, from about 200 to 2800 amino acids long, and are often rich in glycosylation sites and proline residues 13. With these special molecular characteristics, it is likely to participate in cell adhesion and fusion. Although a part of GPCRs interact with G proteins to activate downstream signaling pathways, GPR125 may function through G-protein-independent signaling pathways 46. In our previous research, we found one of the G proteins-Gα13, which was induced by the combined stimulation of RANKL and M-CSF, can negatively regulate osteoclastogenesis through inhibition of the Akt-GSK3β-NFATc1 signaling pathway 25. Thus, there is an intimate relationship between RANKL initiated and GPR-125 guided osteoclastogenesis. However, how RANKL target to GPR-125 or to its downstream G protein, and for which G protein GPR-125 can target to in regulating osteoclastogenesis, it still needs further work in our following research to answer these questions. To explore the possible mechanism by which GPR125 positively regulates osteoclasts, we applied one classic approach to elucidate potential mechanisms. After stimulating with RANKL, we detected the phosphorylation levels of critical proteins of above classic G-protein-independent signaling pathways. MAPKs signaling pathways showed the greatest change between sh-GPR125 group and sh-NC group (**Fig. 6C**). The results showed that p-IKBα proteins and p-P65 were downregulated in sh-GPR125 group compared with sh-NC group (**Fig. 6E**).Though there was no expression level differences for IKBα and P65 proteins between sh-GPR125 group and sh-NC group (**Fig. 6E**), the ratio of p-IKBα/IKBα and p-P65/P65 was obviously downregulated in sh-GPR125 group (**Fig. 6F**), which illustrated that NF-κB signaling pathways was compromised after GPR125 was downregulated. It is reported that the phosphorylated version of IKBα and P65 (p-IKBα and nuclear protein P65) was involved in the classical NF-κB signaling pathways 47. Our results also showed that p-AKT was dramatically decreased in sh-GPR125 group compared with sh-NC group (**Fig. 6E, F**). Collectively, our study has illustrated that GPR125 responds to RANKL stimulation to promote osteoclastogenesis through upregulating AKT and NF-κB signaling pathways to increase the expression of osteoclast genes **(Fig. 7)**. The ligands for the GPCRs have tremendous variation: ions, organic odorants, amines, peptides, proteins, lipids, nucleotides, and even photons are able to mediate their message through these proteins 13. Whichever ligand binds to Gpr125, in our research, it promoted osteosteoclastogenesis with the initiated role of Rankl (Fig. 7). Thus, Gpr125 controlling osteoclasts differentiation in physiological or pathological conditions (i.e. osteoporosis) is regulated by Rankl. Our study suggests that targeting GPR125 may be a novel therapeutic approach for osteolytic diseases.

Though Gpr125 plays an important role in promoting osteoclastogenesis by AKT and NF-κB signaling pathways, there still have some limitation for this research. First, all of these data were based on the physiological state, we have not yet further proved Gpr125's role in osteoclasts under phathological state, which warrants further study. Second, for the signaling pathway research, we did not find which G protein was targeted by Gpr125, and how Rankl regulate Gpr125 in ehancing osteoclastogenesis. However, in our previous research, we found Gα13, a very important G protein, which was induced by the combined stimulation of RANKL and M-CSF, can negatively regulate osteoclastogenesis through inhibition of the Akt-GSK3β-NFATc1 signaling pathway 25.

## Supplementary Material

Supplementary table.

## Abbreviations

GPR125: G-protein 125
RANKL: receptor activator of nuclear factor kappa-B ligand
NF-κB: nuclear factor kappa-B
M-CSF: macrophage colony-stimulating factor
RANK: receptor activator of nuclear factor kappa-B
TRAP: tartrate-resistant acid phosphatase
CTSK: Cathepsin K
GPCR: G-protein coupled receptor
aGPCR: adhesion G-protein coupled receptor
FBS: fetal bovine serum
α-MEM: alpha-minimal essential medium
MBM: mouse bone marrow
qRT-PCR: quantitative reverse transcription polymerase chain reaction
GAPDH: glyceraldehyde-3-phosphate dehydrogenase
IF: immunofluorescence
shRNA: short hairpin RNA
WGA: wheat germ agglutinin
PBS: phosphate buffer solution
AO: acridine orange
