# Supplementary material for: GPR125 positively regulates osteoclastogenesis potentially through AKT-NF-κB and MAPK signaling pathways
Source: Int J Biol Sci. 2022 Mar 6;18(6):2392–405. doi: 10.7150/ijbs.70620 (PMC8990458; doi:10.7150/ijbs.70620)
Supplement: Supplementary file 1 — Supplementary table. [file ijbsv18p2392s1.pdf]

GPR125 positively regulates osteoclastogenesis potentially through AKT-NF-κB and MAPK signaling pathways

Chen-Yi Tang[2,#], He Wang[2,3,#], Yan Zhang[2], Zhongliang Wang[3], Guochun Zhu[2], Abigail McVicar[1], Yi-Ping Li[1,2,*], Wei Chen[1,2,*]

Table S1: primers for qPCR

Gene name	Primer Sequence
Gpr125	Forward: 5'-CACGGTCACCCTGATTTTAAGC-3'
	Reverse: 5'-GCACCTGGGGCTATCCTACTAA-3'
Gapdh	Forward: 5'-GACCACAGTCCATGCCATCAC-3'
	Reverse: 5'-TCCAC CACCCTGTTGCTGTAG-3'
Ctsk	Forward: 5'-GGGCTCAAGGTTCTGCTGC-3'
	Reverse: 5'-TGGGTGTCCAGCATTTCCTC-3'
Pu.1	Forward: 5'-CCCCACACCGGCCTCAGTCACCAG-3'
	Reverse: 5'-CCCCCACGGCCCAGCACCTC-3'
Atp6i	Forward: 5'-CACAGGGTCTGCTTACAACTG-3'
	Reverse: 5'-CGTCTACCACGAAGCGTCTC-3'
Acp5	Forward: 5'- CACTCCCACCCTGAGATTTGT-3'
	Reverse: 5'- CATCGTCTGCACGGTTCTG-3'
